# Regulation of PI3K effector signalling in cancer by the phosphoinositide phosphatases

**DOI:** 10.1042/BSR20160432

**Published:** 2017-02-10

**Authors:** Samuel J. Rodgers, Daniel T. Ferguson, Christina A. Mitchell, Lisa M. Ooms

**Affiliations:** Cancer Program, Monash Biomedicine Discovery Institute and Department of Biochemistry and Molecular Biology, Monash University, Victoria 3800, Australia

**Keywords:** AKT, cancer, phosphoinositide 3-kinase, phosphatidylinositol-3,4,5-trisphosphate, phosphoinositide phosphatases, SGK3

## Abstract

Class I phosphoinositide 3-kinase (PI3K) generates phosphatidylinositol 3,4,5-trisphosphate (PtdIns(3,4,5)*P*_3_) at the plasma membrane in response to growth factors, activating a signalling cascade that regulates many cellular functions including cell growth, proliferation, survival, migration and metabolism. The PI3K pathway is commonly dysregulated in human cancer, and drives tumorigenesis by promoting aberrant cell growth and transformation. PtdIns(3,4,5)*P*_3_ facilitates the activation of many pleckstrin homology (PH) domain-containing proteins including the serine/threonine kinase AKT. There are three AKT isoforms that are frequently hyperactivated in cancer through mutation, amplification or dysregulation of upstream regulatory proteins. AKT isoforms have converging and opposing functions in tumorigenesis. PtdIns(3,4,5)*P*_3_ signalling is degraded and terminated by phosphoinositide phosphatases such as phosphatase and tensin homologue (PTEN), proline-rich inositol polyphosphate 5-phosphatase (PIPP) (INPP5J) and inositol polyphosphate 4-phosphatase type II (INPP4B). PtdIns(3,4,5)*P*_3_ is rapidly hydrolysed by PIPP to generate phosphatidylinositol 3,4-bisphosphate (PtdIns(3,4)*P*_2_), which is further hydrolysed by INPP4B to form phosphatidylinositol 3-phosphate (PtdIns3*P*). PtdIns(3,4)*P*_2_ and PtdIns3*P* are also important signalling molecules; PtdIns(3,4)*P*_2_ together with PtdIns(3,4,5)*P*_3_ are required for maximal AKT activation and PtdIns3*P* activates PI3K-dependent serum and glucocorticoid-regulated kinase (SGK3) signalling. Loss of *Pten, Pipp* or *Inpp4b* expression or function promotes tumour growth in murine cancer models through enhanced AKT isoform-specific signalling. INPP4B inhibits PtdIns(3,4)*P*_2_-mediated AKT activation in breast and prostate cancer; however, INPP4B expression is increased in acute myeloid leukaemia (AML), melanoma and colon cancer where it paradoxically promotes cell proliferation, transformation and/or drug resistance. This review will discuss how PTEN, PIPP and INPP4B distinctly regulate PtdIns(3,4,5)*P*_3_ signalling downstream of PI3K and how dysregulation of these phosphatases affects cancer outcomes.

## Introduction: the PI3K/AKT signalling pathway

**Figure 1:**
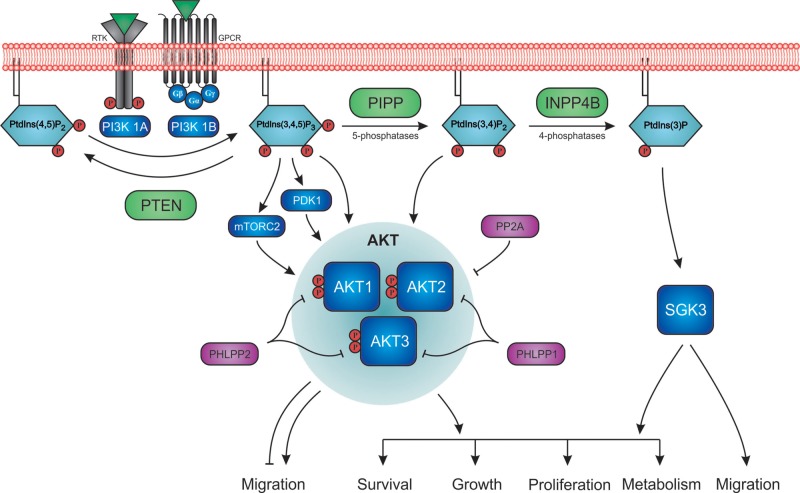
Regulation of PtdIns(3,4,5)*P*_3_ signalling by the phosphoinositide phosphatases Extracellular stimulation of RTKs or GPCRs leads to the recruitment and activation of PI3K1A or PI3K1B respectively, which in turn phosphorylate PtdIns(4,5)*P*_2_ at the D5-position to transiently generate PtdIns(3,4,5)*P*_3_ on the inner leaflet of the plasma membrane. PtdIns(3,4,5)*P*_3_ binds several PH domain-containing proteins such as PDK1, mTORC2 and AKT isoforms (AKT1/2/3). PDK1 and mTORC1 phosphorylate AKT at two distinct phosphorylation sites (e.g. Thr^308^ and Ser^473^ of AKT1 respectively) that promotes its activation. Phosphorylated AKT is dephosphorylated by protein phosphatases PHLPP1/2 and PP2A, which inhibits its activity. PtdIns(3,4,5)*P*_3_ is rapidly dephosphorylated by PTEN to form PtdIns(4,5)*P*_2_, terminating PI3K signalling. Alternatively, PtdIns(3,4,5)*P*_3_ is also dephosphorylated by inositol polyphosphate 5-phosphatases (5-phosphatases) such as PIPP to generate PtdIns(3,4)*P*_2_, which is also required for maximal AKT activation. PtdIns(3,4)*P*_2_ is hydrolysed by inositol polyphosphate 4-phosphatases (4-phosphatases) such as INPP4B to generate PtdIns3*P*, which facilitates phosphorylation and activation of SGK3. AKT and SGK3 activate a number of downstream signalling cascades that regulate cellular processes including cell growth, proliferation, survival, metabolism and migration.

The class I phosphoinositide 3-kinase (PI3K) signalling pathway is a dynamic regulator of physiological and cellular processes including cell proliferation, growth, survival, migration and metabolism. Hyperactivation of PI3K/AKT signalling frequently occurs in human cancers, thus making it an attractive therapeutic target. Class IA PI3Ks are heterodimeric enzymes consisting of a p110α/β/δ catalytic subunit and a p85 regulatory subunit and are directly activated by receptor tyrosine kinases (RTKs). Class IB PI3K heterodimers consist of a p110γ catalytic subunit and a p101 regulatory subunit and are activated downstream of G-protein-coupled receptors (GPCRs). *PIK3CA*, which encodes the p110α subunit of class I PI3K, is frequently mutated or amplified in solid and haematological tumours [1,2]. Class IA or IB PI3Ks are activated upon extracellular stimulation of RTKs or GPCRs, and once activated phosphorylate the D3-position of the inositol ring of phosphatidylinositol 4,5-bisphosphate (PtdIns(4,5)*P*_2_) to transiently generate a pool of phosphatidylinositol 3,4,5-trisphosphate (PtdIns(3,4,5)*P*_3_) at the plasma membrane (Figure 1). PtdIns(3,4,5)*P*_3_ is rapidly dephosphorylated at the D5-position of the inositol ring by inositol polyphosphate 5-phosphatases producing phosphatidylinositol 3,4-bisphosphate (PtdIns(3,4)*P*_2_). Both PtdIns(3,4,5)*P*_3_ and PtdIns(3,4)*P*_2_ facilitate the plasma membrane recruitment of pleckstrin homology (PH)-domain containing proteins such as the serine/threonine kinase AKT [3–5]. Upon phosphoinositide binding, AKT is phosphorylated at Threonine-308 (Thr^308^) within the T-loop region of the catalytic domain by phosphoinositide-dependent kinase 1 (PDK1) and at Serine-473 (Ser^473^) in the C-terminal hydrophobic motif by mammalian target of rapamycin complex 2 (mTORC2), thereby promoting its kinase activity to phosphorylate a diverse spectrum of protein targets [5,6]. PI3K-dependent AKT signalling is inhibited by phosphatase and tensin homologue (PTEN), which hydrolyses PtdIns(3,4,5)*P*_3_ at the D3-position phosphate of the inositol ring to form PtdIns(4,5)*P*_2_ thus directly opposing PI3K. Alternatively, PtdIns(3,4,5)*P*_3_ can be hydrolysed by inositol polyphosphate 5-phosphatases including proline-rich inositol polyphosphate 5-phosphatase (PIPP) to form PtdIns(3,4)*P*_2_, which in turn is degraded by inositol polyphosphate 4-phosphatases such as inositol polyphosphate 4-phosphatase type II (INPP4B) to generate phosphatidylinositol 3-phosphate (PtdIns3*P*), which also terminates PI3K/AKT signalling [7,8].

## AKT has three distinct isoforms

AKT has three highly homologous isoforms (AKT1, AKT2 and AKT3) expressed from distinct genes that are located on separate chromosomes. *AKT1* and *AKT2* transcripts are ubiquitously expressed in human tissues, but *AKT3* expression is more restricted with the highest levels detected in brain, testes, lungs and mammary tissues [9]. Isoform-specific *Akt* knockout mice display distinct physiological phenotypes such as reduced body weight (*Akt1^−/−^*), a diabetic-like phenotype (*Akt2^−/−^*) or impaired brain development (*Akt3^−/−^*) indicating that the three isoforms play non-redundant functional roles [10–15]. All three AKT isoforms contain both a T-loop (Thr^308^) and hydrophobic motif (Ser^473^) and are activated in a similar manner. Following AKT membrane recruitment, co-ordinated phosphorylation of these residues by protein kinases such as PDK1 and mTORC2 promotes AKT activation [5,6]. In fact, AKT is bound by the scaffolding protein IQGAP1 in a protein complex with class I PI3K, PDK1 and several other pathway effectors to facilitate rapid synthesis of PtdIns(3,4,5)*P*_3_ and AKT activation [16]. However, whether the IQGAP1 complex mediates AKT isoform-specific activation remains to be determined. Previously, additional protein kinases have been shown to specifically phosphorylate Thr^308^ or Ser^473^ residues of AKT including DNA-dependent protein kinase (DNA-PK), integrin-linked kinase (ILK) and PI3K that may be implicated in isoform-specific activation [17–20]. Upon its activation, AKT phosphorylates numerous downstream targets including GSK3β, PRAS40, FOXO and p27 [7]. Although many effectors are phosphorylated by all three AKT isoforms, several isoform-specific substrates have also been identified such as palladin that is phosphorylated exclusively by AKT1 and regulates cytoskeletal remodelling [21]. Similarly, regulation of a number of AKT isoform-specific downstream effectors have been identified, including the degradation of nuclear factor of activated T cells (NFAT) mediated by AKT1 and up-regulation of β_1_-integrin by AKT2 that regulates breast cancer cell migration [22,23]. Although they have overlapping roles, there is emerging evidence that the distinct AKT isoforms have specific and sometimes paradoxical functions in cancer, which may be related to differences in their tissue expression, activation states, subcellular localization or substrates and downstream effectors.

## Alterations of AKT isoforms in cancer

There is evidence of AKT dysregulation in some cancers arising from mutations, amplification or hyperactivation of specific AKT isoforms. Somatic AKT mutations occur in up to 5% of human cancers and are clustered in the PH and kinase domains [24]. Although the consequences of most AKT mutations have not been functionally verified, a sporadic E17K hotspot mutation in the PH domain of AKT1 has been identified in breast, colorectal and ovarian cancers that promotes constitutive AKT1 recruitment to the plasma membrane [25]. AKT1^E17K^ is associated with ER-positive breast cancers [26,27]. Patients with breast cancers bearing AKT1^E17K^ mutations exhibit worse outcomes compared with patients with tumours expressing wild-type AKT1 [26]. Furthermore, 16% of AKT1-mutant tumours display no additional alterations involved in disease progression suggesting that AKT1^E17K^ is a potent oncogenic driver [26]. Transgenic expression of AKT1^E17K^ in murine mammary epithelial cells results in mammary hyperplasia and increased oestrogen receptor expression, although these mice do not develop malignant tumours even upon oestrogen exposure [28]. However, knockin of AKT1^E17K^ in PIK3CA^wild-type^ replete MCF-7 luminal breast cancer cells restores anchorage-independent cell growth and xenograft tumour growth comparable to parental MCF-7 PIK3CA^E545K^ cells suggesting that AKT^E17K^ is a *bona fide* oncogene [29]. An E17K mutation in AKT3 was also identified in one case of primary human melanoma, which may have similar functional consequences to AKT1, although this has not been functionally verified [30].

Genetic amplification of AKT isoforms is a relatively uncommon event in cancer. *AKT1* is occasionally amplified in human malignancies, including glioblastoma and a single case of human gastric adenocarcinoma [31,32]. *AKT2* is amplified in cancers such as ovarian (12.2%), breast (2.8%) and pancreatic cancers (10%) and its expression is elevated in pancreatic ductal adenocarcinomas and colorectal cancers [33–37]. *AKT3* is the most amplified isoform in a range of cancers including glioblastoma, melanoma, endometrial and breast cancers [38]. Up-regulation of *AKT3* mRNA and protein expression levels occurs in oestrogen receptor-negative breast cancers and androgen receptor-independent prostate cancer cells, and phosphorylated AKT3^Ser473^ expression is increased in metastatic melanomas [39,40].

Hyperactivation of the PI3K/AKT pathway frequently results from dysregulation of the upstream regulatory proteins, rather than alterations in AKT itself. PDK1 phosphorylates the Thr^308^ residue of AKT as well as other members of the AGC kinase family and is amplified in human breast cancers [41,42]. Phosphorylation of Thr^308^ by PDK1 primes AKT for phosphorylation of its Ser^473^ residue by mTORC2, which is a protein complex made up of the scaffolding protein mLST8, the catalytic subunit mTOR and regulatory proteins including DEP domain-containing mTOR-interacting protein (DEPTOR), Tti1/Tel2, RICTOR and mSin1 [43]. Overexpression of Rictor frequently occurs in human cancers, and *RICTOR* amplification has been identified in breast cancer, residual triple negative breast cancers following neoadjuvant therapy and lung adenocarcinomas with mTORC1/2-inhibitor susceptibility [44–46]. In addition, a D412G mutation in the PH domain of the mSin1 inhibitory subunit of mTORC2 was identified in ovarian cancer, which promotes constitutive mTORC2 activation [47].

More recently, additional protein kinases have been identified that hyperphosphorylate AKT at the Ser^473^/Thr^308^ residues and promote AKT kinase activity in cancer. DNA-PK phosphorylates nuclear AKT at the Ser^473^ region in response to DNA-damage in platinum-resistant ovarian cancer cells where it mediates chemoresistance [17]. In 3T3-L1-GLUT4myc adipocytes, PI3K (p110β/p85α) directly phosphorylates AKT1 at Ser^473^/Thr^308^ and AKT2 residues at Ser^474^ under insulin-stimulated conditions [18]. ILK in complex with RICTOR phosphorylates the Ser^473^ residue of AKT in MDA-MB-231 and MDA-MB-468 breast cancer cells and PC3 prostate cancer cells, where it promotes cell survival and invasion independent of mTORC2 [19,20]. However, expression of murine *Ilk* with point mutations in the putative kinase domain reveals the *in vivo* kinase activity of ILK is dispensable for its function in normal mouse renal development, suggesting it instead serves as an adaptor protein rather than a direct AKT kinase [48]. Phosphorylation of the extreme C-terminal region (Ser^477^/Thr^479^) of AKT1 by CDK2/Cyclin A2 complex primes and promotes AKT1 Ser^473^ phosphorylation, resulting in increased AKT-driven tumour growth *in vivo* [49]. In addition, post-translational modifications of AKT isoforms such as sumoylation or *O-*GlcNAcylation of AKT1, and ubiquitination of AKT1/2 are known to regulate AKT activation and may affect its function in cancer [50–52].

**Figure 2:**
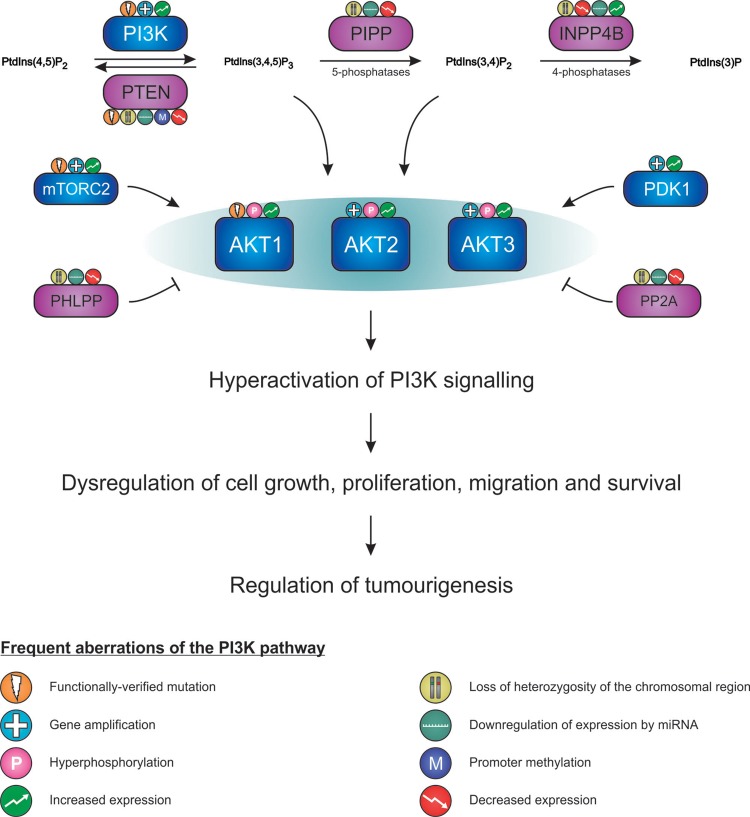
Dysregulation of the PI3K/AKT signalling pathway promotes AKT hyperactivation and tumorigenesis Hyperactivation of PI3K/AKT signalling commonly occurs following dysregulation of the PI3K pathway regulatory proteins including PI3K, AKT, mTORC2, PDK1, PTEN, PIPP, INPP4B, PHLPP and PP2A. Aberration in the function of these proteins can result from mutations, gene amplification, promoter methylation, hyperphosphorylation, LOH, down-regulation by miRNAs or changes in protein and mRNA expression. This leads to disruption of downstream pathway effectors that regulate cell growth, proliferation, migration and survival.

PI3K/AKT signalling may also be increased and sustained in some human cancers due to dysregulation of the protein and lipid phosphatases that modulate the PI3K/AKT signalling pathway (Figure 2). Protein phosphatase 2 (PP2A) complex and PH domain and leucine-rich repeat protein phosphatases 1 and 2 (PHLPP1/2) directly dephosphorylate AKT, thus opposing its phosphorylation-dependent activation (reviewed in [53,54]). PHLPP2 specifically dephosphorylates the hydrophobic Ser^473/472^ motif of AKT1 and AKT3, whereas PHLPP1 dephosphorylates the Ser^474/472^ motif of AKT2 and AKT3 [55,56]. PHLPP1/2 expression is frequently decreased in human cancers such as colon, breast, ovarian, prostate and hepatocellular carcinoma (HCC) [54]. Loss of heterozygosity (LOH) of the chromosomal region (18q21.33) to which PHLPP1 maps occurs in colon cancers, and LOH of the PHLPP2 locus (16q22.3) is observed in HCCs, Wilms’ tumours and breast, ovarian and prostate cancers [57–62]. The subunits of PP2A (e.g. PR65/A, B56) also show decreased expression in cancers such as melanoma, acute myeloid leukaemia (AML), breast cancer and colorectal cancer [53]. In addition, the spatio-temporal regulation of PtdIns(3,4,5)*P*_3_ and PtdIns(3,4)*P*_2_ is essential for recruiting AKT to the plasma membrane to be phosphorylated by protein kinases. The phosphoinositide phosphatases such as PTEN, PIPP and INPP4B dynamically regulate PtdIns(3,4,5)*P*_3_ and PtdIns(3,4)*P*_2_ levels, and play prominent roles in human cancers and their function will be further discussed below. Thus, the PI3K/AKT pathway is a complex network of proteins and phosphoinositides that can be altered at many different points leading to dysregulation of the signalling axis.

## Divergent functions of AKT isoforms in cancer

AKT is considered a *bona fide* oncogene in human cancers, yet disruption of individual AKT isoforms reveals distinct and opposing roles in tumorigenesis (Table 1). *Akt1* or *Akt2* knockout in a viral oncogene-induced mouse model of lung cancer demonstrated that *Akt1*-ablation inhibited, whereas *Akt2*-ablation enhanced lung tumour initiation, highlighting their functionally diverse roles [63]. In a similar manner, transgenic expression of AKT1 accelerates the tumour incidence of *PyMT* mammary tumour mice, while AKT2 transgenic expression had no effect on tumour latency [64]. However, transgenic mammary expression of AKT1 or AKT2 alone in wild-type mice is insufficient to promote *de novo* tumour formation [64,65]. In contrast, hepatic *Akt1* knockout in an *Akt2*-null murine model triggers a FOXO-dependent inflammatory response leading to spontaneous HCC, which was not observed with hepatic knockout of *Akt1* or *Akt2* alone, suggesting a novel co-operative and potentially tumour-suppressive effect of AKT1/2 in hepatic tissue [66]. In triple negative breast cancers, increased AKT3 expression is prevalent and may be driven by gene amplification [67,68]. shRNA-mediated knockdown of *AKT1, AKT2* or *AKT3* in triple negative breast cancer cells revealed that AKT3 is preferentially required for 3D tumour spheroid growth and *in vivo* xenograft tumour growth through regulation of the cell-cycle inhibitor p27, whereas knockdown of *AKT1* and *AKT2* had little effect on tumour growth [67]. Furthermore, AKT3 depletion sensitizes triple negative breast cancer cells to the pan-AKT inhibitor GSK690693 [67]. Similarly, AKT3 is up-regulated in T47D luminal breast cancer cells in response to the AKT inhibitor MK2206 that confers resistance to MK2206, and *AKT3* depletion in these cells selectively increases sensitivity to MK2206 treatment whereas *AKT1* or *AKT2* depletion has no effect [69]. *AKT3* mRNA and protein expression is also increased in prostate tumours, and overexpression of AKT3 promotes cell proliferation in a range of prostate cancer cell lines [70].

**Table 1:** Divergent functions of AKT isoforms in mouse models of cancer

	AKT isoform expression	AKT1	AKT2	AKT3	References
**Global knockout**	↓ ^1^	Reduced body weight	Diabetic-like phenotype	Impaired brain development	[10–12,15]
**Tumour latency**	↑ ^2^	Reduces (mammary)	No effect (mammary)	Not reported	[64,65]
	↑ ^3^	Reduces (melanoma)	Not reported	Not reported	[79]
	↓ ^1^	Increases (lung)	Reduces (lung)	Minimal effect (lung)	[63]
	↓ ^4^	Reduces (hepatic)			[66]
**Tumour incidence**	↑ ^3^	No effect (glioma)	Increases (glioma)	Increases (glioma)	[38]
	↓ ^1^	No effect (lung)	Increase (lung)	Minimal effect (lung)	[63]
**Tumour metastasis**	↑ ^2^	Reduces (mammary)	Increases (mammary)	Not reported	[64,65]
	↑ ^3^	Increases (melanoma)	Not reported	Not reported	[79]

^1^Global knockout. ^2^Tissue-specific transgene. ^3^RCAS-TVA system. ^4^Hepatic *Akt1^−/−^* and global *Akt2^−/−^*.

The phenotype of *Akt3* knockout mice indicates that AKT3 function is critical in brain tissue, thus, perhaps not surprisingly, AKT3 plays a significant role in human gliomas. In primary murine astrocytes with mutant PTEN/p53/EGFR alleles, *Akt3*-ablation specifically inhibited anchorage-independent cell growth while *Akt1*- or *Akt2*-ablation had no effect [71]. Similarly in a PDGFB-driven mouse model of low-grade glioma, transgenic expression of AKT2 or AKT3 but not AKT1 greatly accelerated tumour formation [38]. Strikingly, RNA microarray analysis revealed that transgenic AKT3 expression enriches expression of genes associated with DNA damage response, which mediates DNA repair and resistance to radiotherapy and chemotherapy treatments suggesting that increased AKT3 expression may promote malignancy [38]. However, in a separate study, AKT3 overexpression reduced cell-cycle progression and cell survival in human glioblastoma cell lines, and increased the tumour survival of mice with orthotopic injection of glioblastoma cells [72]. Furthermore, increased *AKT3* mRNA levels were associated with increased patient survival and lower grade glioblastomas suggesting a more favourable outcome for these patients, whereas *AKT1* and *AKT2* expression was increased in higher grade tumours [72].

In addition to their divergent functions in tumour growth and maintenance, AKT isoforms have distinct functions in regulating cell migration and cancer metastasis that are highly context and cell-type specific. Transgenic overexpression of constitutively active AKT1 and AKT2 in oncogene-driven mouse models of breast cancer have revealed their opposing effects on cell migration and tumour metastasis, whereby AKT1 inhibits but AKT2 promotes the establishment of metastatic lesions [64,65,73]. *In vitro* studies in breast cancer cell lines suggest that AKT1-mediated degradation of the pro-invasion transcription factor NFAT and the tumour-suppressor tuberous sclerosis complex 2 (TSC2) decreases, whereas AKT2-mediated up-regulation of pro-invasive β1-integrin promotes cell migration [22,23,74]. In addition, the actin-bundling protein palladin is specifically phosphorylated at Ser^507^ and activated by AKT1 leading to an inhibition of cell migration mediated via cytoskeletal remodelling [75]. In contrast, AKT2 promotes palladin stability and mRNA up-regulation via unknown mechanisms [76]. Phosphorylation of Rho-GTPase by AKT1 in inflammatory breast cancer cells is critical for promoting caveolin-1-mediated migration suggesting that AKT1 conversely promotes migration in this cellular context [77].

In PC-3 prostate cancer cells, siRNA-mediated knockdown of *AKT1* inhibited cell migration and cell adhesion, whereas *AKT2* knockdown promoted cell migration suggesting that AKT1 has a pro- and AKT2 has an antimigratory role in prostate cancer, in contrast with their functions in breast cancer [78]. Similarly, transgenic expression of constitutively active AKT1 in BRAF^V600E^/Cdkn2a^Null^ non-metastatic melanoma model mice induces metastatic lesions in the brain and lung [79]. In MDA-MB-231 and MCF-7-Ras breast cancer cells, which have more stem-like properties, AKT1 inhibition has a more prominent effect than AKT2 inhibition in reducing the cancer cell stem phenotype, as reflected by reduced mesenchymal-epithelial transition (MET) and expression of epithelial-like markers [80]. As epithelial–mesenchymal transition (EMT) is a critical process in metastatic invasion, AKT1 induction of stem properties may confer an increase in invasive and metastatic potential of stem-like tumour cells. Interestingly, *Akt3*-ablation in mutant PTEN/p53/EGFR murine astrocytes inhibited cell migration whereas *Akt1 and Akt2* ablation had no effect [71]. However, in vascular tumour cells *AKT3* depletion increases whereas *AKT1* depletion decreases sprouting angiogenesis and wound healing capacity, suggesting that AKT3 conversely inhibits vascular tumour growth and migration [81].

AKT displays a range of isoform-specific functions in different tissues, yet the explanation for such a divergence in functions is poorly understood. These distinct functions are likely to be highly context-specific and affected in part by expression levels, subcellular localization and/or the unique interactome of the different isoforms. Other kinase families such as the protein kinase C (PKC) isozymes have overlapping and opposing functions in human cancers similar to AKT, suggesting that divergent kinase functions are likely to mediate a homoeostatic balance of cellular pathways that are often exploited in human malignancies [82]. As clinical trials with pan-AKT inhibitors have shown limited success in cancer treatment this far, perhaps a greater understanding of the isoform-specific effects of AKT may assist in the development of more targeted AKT isoform therapeutic strategies. Moreover, increasing our understanding of AKT regulatory enzymes, particularly the phosphoinositide phosphatases, may elucidate additional contributing factors for isoform-specific signalling. The inositol polyphosphate phosphatases including PTEN, PIPP and INPP4B regulate PtdIns(3,4,5)*P*_3_ and PtdIns(3,4)*P*_2_ levels and thus modulate AKT activation. These lipid phosphatases were initially predicted to be tumour suppressors whereby loss of expression would increase PI3K/AKT signalling and tumour growth and progression thereby leading to a worse prognosis. However, this review will discuss their roles in regulating isoform-specific AKT functions, and their potential to play highly dynamic and complex roles in cancer biology beyond a conventional tumour suppressor function.

## Regulation of PtdIns(3,4,5)*P*_3_ signalling by phosphoinositide phosphatases

### PTEN

PTEN is a well-established tumour suppressor and its function is lost in a wide spectrum of human cancers via multiple mechanisms including sporadic mutations, deletions, transcriptional silencing, protein instability or subcellular mislocalization (reviewed in [83,84]). *PTEN* is one of the most frequently mutated and down-regulated tumour suppressive genes in human cancer [83]. Single germ line mutations in *PTEN*are sufficient to predispose individuals to PTEN hamartoma tumour syndromes (PHTS) that result in tumour-like lesions throughout the body and an increased risk of developing malignant tumours [85]. *Pten*^−/−^ mice die embryonically, but *Pten* haploinsufficient mice in part recapitulate PTEN-deficient human cancers, and exhibit widespread neoplasia and hyperplasia in multiple tissues [86–88]. Functionally, PTEN is a dual specificity protein phosphatase that dephosphorylates p-tyrosine, -serine and -threonine residues as well as a lipid phosphatase that hydrolyses the D3-position phosphate from the inositol head group of PtdIns(3,4,5)*P*_3_. PTEN’s tumour suppressor function was first characterized via its phosphoinositide phosphatase activity, whereby hydrolysis of PtdIns(3,4,5)*P*_3_ by PTEN directly opposes PI3K signalling activity [89–91]. Thus, PTEN loss drives PI3K/AKT hyperactivation. The phosphoprotein phosphatase function of PTEN has been linked to cancer signalling via dephosphorylation of protein targets such as focal adhesion kinase (FAK), insulin receptor substrate 1 (IRS-1), c-SRC or PTEN itself, all of which regulate tumorigenesis [92–95]. However, studies *in vitro* and *in vivo* have confirmed that PTEN phosphoinositide phosphatase activity plays a more predominant tumour suppressor role than the phosphoprotein activity [96,97]. For example, mice with single allele knockin of either the catalytically inactive (C124S) mutant lacking both protein and lipid phosphatase activity or a lipid phosphatase inactive (G129E) PTEN mutant, display similar tumour spectra to each other, but show accelerated tumorigenesis compared with *Pten^+/−^* mice [97]. PTEN mutant proteins heterodimerize with wild-type PTEN protein thereby disrupting PTEN function in a dominant negative manner [97]. Critically, AKT hyperactivation resulting from loss of PTEN lipid phosphatase function is the prominent oncogenic driving force in PTEN-deficient cancers.

The molecular mechanisms by which specific AKT isoforms mediate tumorigenesis downstream of PTEN-loss have not been well characterized. Initial reports suggested a prominent role for AKT1 in PTEN-deficient cancers. Strikingly, *Akt1* ablation in *Pten^+/-^* mice prevented the onset of neoplasia in endometrial, prostate and thyroid tissues, and reduced the incidence of intestinal polyps and high-grade neoplastic lesions in the adrenal medulla (Table 2) [98]. Conversely, knockout of *Akt2* in *Pten*^+/−^ mice had no significant effects on neoplastic growth in most tissues except the thyroid gland, where the inhibition of neoplastic incidence was comparable to *Akt1^−/−^;Pten^+/−^* mice (Table 2) [99]. Examination of the relative expression of *AKT1*and *AKT2*in these murine tissues revealed that the thyroid gland was the only tissue where AKT2 expression was higher than that of AKT1, suggesting a model whereby the onset of PTEN-deficient cancer is preferentially driven by AKT1, except in tissues where AKT2-enrichment is sufficient to co-operatively drive neoplasia.

**Table 2:** PI3K signalling effector regulation following dysregulation of PTEN, PIPP or INPP4B in melanoma, breast, prostate, thyroid and colorectal cancers

Cancer type	PTEN	PIPP	INPP4B (tumour suppressor function)	INPP4B (oncogenic function)
**Breast cancer**	**AKT2**	**AKT1**	**AKT**	**SGK3**
	Knockdown of *AKT2* in *PTEN*-deficient breast cancer cells reduces 3D spheroid growth [100].	Knockdown of *AKT1* rescues cell migration defect in *PIPP*-deficient breast cancer cells [105].	*INPP4B* knockdown promotes AKT-mediated breast cancer cell growth and proliferation [121,129].	*INPP4B* knockdown reduces SGK3-mediated cell growth and proliferation [144].
**Melanoma**	**AKT**	**AKT**	**AKT**	**SGK3**
	*PTEN* knockdown in melanocytes enhances AKT-mediated cell growth [107].	PIPP overexpression in melanoma cells reduces AKT-mediated cell proliferation and survival [107].	*INPP4B* knockdown promotes AKT-mediated melanoma cell growth and proliferation [133].	INPP4B overexpression promotes SGK3-mediated cell growth and proliferation [143].
**Prostate cancer**	**AKT1/AKT2**	Not reported.	**AKT**	Not reported.
	*Akt1* ablation prevents prostate tumour onset in *Pten^+/−^* mice [98].		*INPP4B* knockdown promotes AKT-mediated prostate cancer cell growth and proliferation [130].	
	Knockdown of *AKT2* in *PTEN*-deficient prostate cancer cells reduces 3D spheroid growth [100].			
**Thyroid cancer**	**AKT1/AKT2**	Not reported.	**AKT2**	Not reported.
	*Akt1* or *Akt2* ablation prevents thyroid tumour onset in *Pten^+/−^* mice [98].		*Inpp4b* ablation in *Pten^+/−^* mice promotes AKT2-dependent thyroid tumour growth [126].	
**Colorectal cancer**	**AKT2**	Not reported.	Not reported.	**AKT/SGK3**
	PTEN loss is required for metastasis of colorectal cancer cells overexpressing AKT2 [101].			INPP4B overexpression promotes AKT- and SGK3-mediated cell growth and proliferation [128].

However, other findings challenge the dispensability of AKT2 in the progression of *PTEN*-deficient solid tumours including prostate and breast cancer and glioblastoma [100]. Inducible shRNA knockdown of *AKT1* or *AKT2* in *PTEN*-deficient prostate cancer cells inhibited the formation of 3D spheroids, suggesting that both AKT isoforms may be required for initial tumour growth. In contrast, induction of *AKT2* but not *AKT1* silencing after 1 week caused widespread apoptosis and compromised cell morphology leading to complete disruption of spheroid architecture, which was recapitulated upon treatment of spheroids with an AKT2-specific inhibitor, suggesting that AKT2 plays a dominant role in 3D tumour survival and progression (Table 2) [100]. *AKT2* silencing had a similar effect on *PTEN*-deficient breast cancer and glioblastoma cell models, whereby *AKT2* knockdown caused regression of 3D spheroid growth comparable to prostate cancer models (Table 2) [100]. Importantly, induction of *AKT1* knockdown slowed xenograft tumour growth, whereas *AKT2* knockdown resulted in a striking regression of tumour size suggesting decreased tumour survival capacity [100]. The co-operativity between AKT2 and PTEN-deficiency was further demonstrated in colon cancer, where loss of PTEN function was required for enhanced liver metastasis of intrasplenically injected colorectal cancer cells overexpressing AKT2 (Table 2) [101]. These studies highlight the differential yet indispensable roles of AKT1 and AKT2 in *PTEN*-deficient cancer development, suggesting that AKT1 may drive the initial establishment of solid tumours whereas AKT2 may be intrinsic to tumour maintenance and survival.

### PIPP

PIPP (INPP5J, Pib5pa, PtdIns(4,5)*P*_2_ 5-phosphatase A) has recently been demonstrated to act as a putative tumour suppressor in breast cancer and also as a regulator of AKT1-dependent breast cancer metastasis. PIPP is one of the ten mammalian inositol polyphosphate 5-phosphatases that hydrolyses the D5-position phosphate from the inositol ring of PtdIns(4,5)*P*_2_, PtdIns(3,4,5)*P*_3_, inositol-1,4,5-trisphosphate (Ins(1,4,5)*P*_3_) and inositol-1,3,4,5-tetrakisphosphate (Ins(1,3,4,5)*P*_4_) [102,103]. In addition to the conserved 5-phosphatase domain, PIPP also contains N- and C-terminal proline-rich domains containing six RSXSXP 14–3-3 ζ-binding motifs and a SKICH domain C-terminal to the 5-phosphatase domain, which mediates its constitutive localization to the plasma membrane in quiescent and epidermal growth factor (EGF)-stimulated cells [104]. Although both PtdIns(3,4,5)*P*_3_ and PtdIns(3,4)*P*_2_ are required for maximal AKT activation, a number of studies have revealed that PIPP regulates AKT activation and consequently the phosphorylation of downstream effectors including GSK3β, PRAS40, 4E-BP1 and p70 S6 kinase [105–107]. Murine knockout of *Pipp* in all tissues results in no overt phenotype at 4 months of age and does not lead to *de novo* tumour formation [105]. However, *Pipp* ablation in an MMTV-*PyMT* mouse model of breast cancer promotes mammary tumour initiation and growth resulting in larger tumours compared with mice expressing *Pipp. PyMT;Pipp*^−/−^ mice also exhibit increased AKT^Ser473^ phosphorylation in both hyperplastic foci and primary mammary tumours suggesting that *Pipp* loss enhances oncogene-driven breast cancer initiation and progression via regulating PI3K/AKT signalling.

Paradoxically, despite promoting the formation of larger mammary tumours, *Pipp* ablation in the *PyMT* mouse model resulted in reduced numbers of lung metastases [105]. Moreover, *Pipp*-deficient mammary cancer cells exhibited reduced cell migration and invasion *in vitro*, a defect rescued by the shRNA-mediated knockdown of *Akt1* but not *Akt2* (Table 1) [105]. This is consistent with the established role for AKT1 in inhibiting and AKT2 in promoting breast cancer cell migration and metastasis [22,108]. There is no evidence that loss of *Pipp* results in differential AKT isoform activation [105]. *AKT1* mRNA is the major isoform expressed in murine mammary tumour cells. However, AKT1 and AKT2 are equally expressed in a number of ER-negative human breast cancer cell lines including MDA-MB-231 cells in which *PIPP* shRNA knockdown also reduced cell migration and expression of AKT1 downstream targets suggesting that the impaired migration is not simply due to differences in AKT isoform levels [105,109]. Alternatively, PIPP regulation of AKT1-dependent cell migration may reflect differences in AKT isoform subcellular localization. AKT1 localizes to the cytoplasm in a number of human breast cancer cell lines, whereas AKT2 is present in mitochondria and the cytoplasm and AKT3 exhibits a nuclear and nuclear membrane distribution [109]. Further studies are required to fully elucidate the complex molecular mechanisms by which phosphoinositide signalling regulates AKT isoform-specific cell migration and metastasis.

*PIPP* is reported to be one of the ten highest ranked genes for predicting outcomes in human breast cancer and therefore understanding its exact role in regulating mammary tumorigenesis and metastasis is of particular importance [110]. The *INPP5J* gene is located on chromosome 22q12 and allelic loss of this region occurs in ~30% of breast cancers [111–113]. Furthermore, reduced *PIPP* copy number has been reported in 15–20% of primary melanomas and melanoma cell lines, and PIPP expression is epigenetically suppressed by HDAC2 and -3-mediated histone hypoacetylation in melanoma cell lines [107]. Higher *PIPP* expression in breast cancer correlates with a better prognosis, defined as no development of distant metastases within 5 years of diagnosis, whereas lower *PIPP* mRNA expression predicts for reduced relapse-free and overall survival [105,114]. However, this decrease in survival does not appear to be consistent with the observation that *Pipp* ablation reduces mammary carcinoma metastasis in MMTV-*PyMT* mice [105]. There are several possible explanations for this apparent paradox. Firstly, although *Pipp* loss significantly reduces mammary carcinoma metastasis, all mice still develop lung metastases in this particular oncogene-driven murine model [105]. As *Pipp* ablation promotes cell proliferation, metastatic *PIPP*-deficient cells may have a proliferative advantage and facilitate secondary tumour establishment and growth at distant sites. Secondly, PIPP regulates cell migration in an AKT1-dependent manner. Expression of both *PIPP* and *AKT1* was reduced in a subset of human breast cancers and it is interesting to speculate that hyperactivated AKT2 in these tumours may promote metastasis leading to a poorer outcome [105] although this has yet to be shown.

Studies in melanoma cell lines and xenografts have revealed that PIPP also acts as a potential tumour suppressor in melanoma. Transient overexpression of PIPP resulted in decreased proliferation, survival and AKT activation in melanoma cell lines (Table 2) [107]. Additionally, overexpression of PIPP in the ME1007 melanoma cell line resulted in reduced xenograft tumour growth [107]. Accordingly, shRNA knockdown of *PIPP* promoted anchorage-independent cell growth of cultured melanocytes [107], similar to the results observed with *PIPP* shRNA in breast cancer cell lines [105]. However, overexpression of PIPP in the MEL-FH melanoma cell line decreased cell migration [115 ]. Interestingly, expression of constitutively active AKT1 promoted metastasis in a murine melanoma model [79] in contrast with the reduced metastasis observed in murine mammary cancer models [64,65,73]. Although the effects of *PIPP* loss on melanoma cell migration and metastasis have not been reported, it is interesting to speculate that this may lead to increased cell migration and metastasis via AKT1 activation.

AKT1 exhibits a cell type-specific role in regulating cell migration in different cancer cells. Knockdown of *AKT1* decreases cell migration in lung and ovarian cancer cells [116,117] but increases cell migration in endometrial and breast cancer cells [105,118]. Conversely, expression of constitutively active AKT1 impairs breast cancer cell migration [22,23,75] but promotes invasion of pancreatic carcinoma and fibrosarcoma cells [119,120]. Therefore, it will be interesting to explore the effects of *PIPP* loss on tumour cell invasion and metastasis in other cancers.

Interestingly, PIPP expression positively correlates with PTEN expression in primary human melanomas, with ~35% of PTEN-null melanomas exhibiting PIPP deficiency [107]. Co-expression of exogenous PIPP and PTEN in a melanoma cell line further decreased pAKT^Ser473^ compared with either phosphatase alone [107]. Conversely, knockdown of both *PIPP* and *PTEN* resulted in increased AKT phosphorylation compared with knockdown of either phosphatase alone suggesting that combined loss of *PIPP* and *PTEN* may additively hyperactivate PI3K/AKT signalling in melanoma cells [107], consistent with the contention that PIPP and PTEN play non-redundant roles in regulating PtdIns(3,4,5)*P*_3_-dependent signalling. However, knockdown of both *PIPP* and *PTEN* may trigger senescence in cultured melanocytes under anchorage-independent conditions, although a proportion of double knockdown cells may evade senescence and form significantly larger colonies [107]. A similar phenotype was observed in human mammary epithelial cells with shRNA knockdown of both *PTEN* and the inositol polyphosphate 4-phosphatase *INPP4B* [121].

### INPP4B

INPP4B together with INPP4A are members of the mammalian inositol polyphosphate 4-phosphatase family. INPP4A and INPP4B share 37% sequence homology and contain an N-terminal C2 domain(s), a PEST sequence and an N-terminal dual specificity 4-phosphatase domain [122,123]. INPPB preferentially displays catalytic activity towards PtdIns(3,4)*P*_2_, but hydrolyses several other lipid species *in vitro* including PtdIns(4,5)*P*_2_, PtdIns(3,4,5)*P*_3_, inositol-1,3,4-trisphosphate (Ins(1,3,4)*P*_3_) and Ins(3,4)*P*_2_ [121,124–126]. Additionally, INPP4B displays intrinsic p-tyrosine, -serine and -threonine phosphatase activity [127,128]. INPP4B was characterized as an inositol polyphosphate 4-phosphatase that preferentially dephosphorylates plasma membrane-bound PtdIns(3,4)*P*_2_ at the D4-position of the inositol ring to form PtdIns3*P* [121,124,125]. As both PtdIns(3,4)*P*_2_ and PtdIns(3,4,5)*P*_3_ are required for AKT recruitment to the plasma membrane and maximal AKT activation, INPP4B was predicted to act as a tumour suppressor by inhibiting PI3K/AKT signalling. Indeed, INPP4B tumour suppressor function was initially identified in breast cancer. *INPP4B* mRNA expression is lost in a cohort of basal-like breast cancers and its reduced expression is associated with higher tumour grade and worse survival [121,129]. LOH of the gene region of *INPP4B*(4q31.21) occurs in basal-like breast tumours (55.6%), ovarian cancers (39.8%) and melanomas (21.6%) [121]. *INPP4B* shRNA knockdown in breast cancer cell lines increased cell proliferation, motility, anchorage-independent cell growth, xenograft tumour growth and disrupted mammary acini morphology in an AKT-dependent manner (Table 2) [121,129]. Interestingly, INPP4B protein expression is frequently lost in primary human PTEN-null breast tumours [129], and *PTEN* depletion in mammary epithelial cells phenocopies the changes in cell proliferation, motility and AKT activation following *INPP4B* depletion [121]. However concomitant shRNA-mediated knockdown of *INPP4B* and *PTEN* decreased cell proliferation and anchorage-independent cell growth compared with control cells, and increased cellular senescence which was rescued upon shRNA knockdown of *p53* [121]. Colonies that formed under anchorage-independent cell growth conditions in *INPP4B/PTEN* knockdown cells were larger than *INPP4B* or *PTEN* single knockdown colonies, suggesting that depletion of both *INPP4B* and *PTEN* can enhance cell growth in rare events in a manner similar to dual *PTEN/PIPP* knockdown in melanoma cell lines [107,121].

In addition, examination of INPP4B function in prostate cancer has supported its role as a tumour suppressor. Loss of *INPP4B* expression in prostate cancers is associated with reduced time for biochemical recurrence and poorer outcomes [130,131]. *INPP4B* shRNA knockdown in LNCaP prostate cancer cell lines increased cell proliferation and AKT activation, whereas its ectopic expression in PC-3 prostate cancer cells decreased *in vivo* stromal invasion in chick–embryo models (Table 2) [130,132]. Similarly, INPP4B protein expression is progressively lost in more advanced stages of human melanocytic tumours, and its shRNA-mediated knockdown in melanoma cell lines enhanced AKT^Ser473^ phosphorylation, proliferation, migration and *in vivo* tumour growth [133]. Collectively, these findings support a model whereby INPP4B functions as a tumour suppressor by negatively regulating PtdIns(3,4)*P*_2_-dependent AKT signalling.

*In vivo* depletion of *Inpp4b* in mice is not sufficient to drive spontaneous tumorigenesis *per se* as *Inpp4b^−/−^* mice are viable with a normal lifespan and no evidence of tumour development up to 2 years of age, although mice exhibit decreased bone mass and osteoporosis from 8 weeks of age [125,126,134]. This is in contrast with *Pten^+/−^* mice that develop hyperplasia and in turn cancer in multiple organs from an early age [86, 87, 88]. Expression of both INPP4B and PTEN is frequently lost in thyroid and endometrial cancers, suggesting a co-operative tumour suppressor function for both enzymes. Consequently, *Inpp4b^−/−^* mice were crossed with *Pten^+/−^* mice to examine the co-operative tumour suppressor function of *INPP4B* in the context of *PTEN* haploinsufficiency. Strikingly, *Inpp4b^−/−^;Pten^+/−^* mice developed aggressive thyroid tumours resembling human follicular variant papillary thyroid carcinoma (FV-PTC), which was not observed in *Pten^+/−^* mice, leading to reduced survival. Furthermore, *Akt2^−/−^;Inpp4b^−/−^;Pten^+/−^* mice exhibited no overt FV-PTC phenotype and showed an improved lifespan, whereas *Akt1^−/−^;Inpp4b^−/−^;Pten^+/−^* were comparable to *Inpp4b^−/−^;Pten^+/−^* mice, suggesting that AKT2 drives *Inpp4b/Pten*-deficient thyroid tumorigenicity (Table 2) [126]. Indeed, this suggests that INPP4B, like PTEN, preferentially regulates AKT2 activation in thyroid tissue in an isoform-dependent signalling model. INPP4B but not PTEN co-localizes with AKT2 and PIK3C2α on early endosomes of thyroid cancer cells where INPP4B negatively regulates PIK3C2α-mediated AKT2 signalling through PtdIns(3,4)*P*_2_ hydrolysis [134]. An independent report showed that PTEN binds to PtdIns3*P*-positive endocytic vesicles along microtubules where it prevents AKT activation through its action on vesicular PtdIns(3,4,5)*P*_3_ hydrolysis [135]. INPP4B together with VPS34 was postulated to dynamically regulate PtdIns3*P* on endocytic vesicles to mediate PTEN recruitment and although this has not been shown experimentally, it suggests an endosomal function for INPP4B signalling. INPP4B can also directly dephosphorylate PtdIns(3,4,5)*P*_3_ in *Pten*-null thyroid tissue and concomitant loss of *Inpp4b* and *Pten* promoted a striking increase in PtdIns(3,4,5)*P*_3_ levels [126]. This analysis suggests in some contexts that INPP4B is a direct regulator of PtdIns(3,4,5)*P*_3_, which is predicted to act as the last line of defence against deleterious PtdIns(3,4,5)*P*_3_ accumulation in *PTEN*-deficient thyroid cancer cells.

INPP4B can directly degrade PtdIns(3,4)*P*_2_ signals by dephosphorylating the inositol head group, yet recent studies suggest that INPP4B may in other contexts increase PtdIns(3,4,5)*P*_3_ levels [128, 136]. TAPP1/2 proteins bind to PtdIns(3,4)*P*_2_ and drive a negative feedback loop that recruits inhibitory PI3K-signalling proteins such as PTPL-1 to decrease PtdIns(3,4,5)*P*_3_ production [136,137]. Degradation of PtdIns(3,4)*P*_2_ by INPP4B was postulated to reduce this TAPP1/2-mediated feedback and thus conversely promote PtdIns(3,4,5)*P*_3_ accumulation [136]. INPP4B has also been shown to indirectly up-regulate PtdIns(3,4,5)*P*_3_ through PTEN destabilization. In colon cancer cell lines, INPP4B binds and dephosphorylates the C-terminal tail region of PTEN leading to PTEN degradation and thereby an increase in PtdIns(3,4,5)*P*_3_ and subsequently PI3K signalling activation [128]. However, this apparent inhibition of PTEN function is in contrast with previous findings, which suggests that non-phosphorylated PTEN displays increased lipid phosphatase activity [138,139]. Thus, the consequences of post-translational modifications by INPP4B on PTEN catalytic activity require further examination. Nonetheless, INPP4B overexpression promoted anchorage-independent cell growth in FHC colon epithelial cells, cell proliferation in SW620 and HT-29 colon cancer cells, and *INPP4B* shRNA knockdown in HTC116 colon cancer cells reduced murine xenograft tumour size [128]. Therefore, despite INPP4B tumour suppressor function being reported *in vivo* and *in vitro* in various cancers, there is emerging evidence that INPP4B also plays a paradoxical oncogenic role in certain other cancer contexts.

The recent emergence of serum and glucocorticoid-regulated kinase (SGK3) as an oncogenic effector in *PIK3CA*-mutant breast cancer cells independent of AKT [140] has led to the examination of INPP4B as a mediator of PI3K/SGK3 signalling. SGK3 is phosphorylated and activated upon binding of its PX domain to endosomal PtdIns3*P* [141]. Treatment of U20S cells with class I PI3K inhibitors (GDC-0941 or BKM120) reduced SGK3 phosphorylation up to 40% in a dose-dependent manner, suggesting that SGK3 is regulated downstream of class I PI3Ks [142]. In colon cancer cells, INPP4B-mediated degradation of PTEN promoted tumour growth, proliferation and co-operatively enhanced AKT and SGK3 activation downstream of PI3K (Table 2) [128]. However, as INPP4B generates a membrane-bound pool of PtdIns3*P*, INPP4B was predicted to trigger SGK3 activation through hydrolysis of plasma membrane-bound PtdIns(3,4)*P*_2_. Indeed, high INPP4B protein expression in fresh melanoma isolates and melanoma cell lines was associated with high pSGK3^T320^ levels [143]. *INPP4B* shRNA knockdown attenuated melanoma cell proliferation and xenograft tumour growth, whereas INPP4B overexpression enhanced cell proliferation and promoted melanocyte anchorage-independent cell growth, driven by INPP4B-mediated activation of SGK3 in an AKT-independent manner (Table 2) [143]. In breast cancer cells, increased SGK3 phosphorylation was associated with increased INPP4B expression, as well as *PIK3CA* and *PTEN* mutations [144 ]. shRNA knockdown of *INPP4B* in MCF-7 and ZR-75-1 breast cancer cells, which express high levels of SGK3, reduced anchorage-independent cell growth, cell migration, 3D colony formation and mouse xenograft tumour growth as well as inhibiting IGF-1-stimulated SGK3 phosphorylation (Table 2) [144]. Thus in cell lines with high SGK3 expression, INPP4B may provide a molecular gateway to the PI3K/SGK3 signalling axis that diverges from the canonical PI3K/AKT signalling pathway. SGK3 co-localizes with EEA1 at early endosomes and does not exhibit a plasma membrane distribution [142 ]. Given that INPP4B localises to early endosomes in thyroid cancer cells, these findings provide further evidence of a potential endosomal function for INPP4B in particular cancers.

The complexity of INPP4B function is also highlighted in AML. Several studies have demonstrated increased INPP4B expression, which leads to chemotherapeutic resistance and poor patient outcomes [145–147]. Increased INPP4B expression was observed in a subset of AML cases associated with reduced therapeutic response, shorter event free and overall survival and was an independent biomarker of patient prognosis [145,146]. Induction of INPP4B in AML cells promoted cell proliferation, survival and desensitization to chemotherapeutic treatment *in vivo* and *in vitro* [145,146]. Conversely, siRNA knockdown of *INPP4B*sensitized AML cells to chemotherapeutic treatment, by inhibiting the activation of several DNA repair proteins including ATM and BRCA1 [147]. However, ectopic expression of a catalytically inactive INPP4B mutant yielded contrasting effects on the therapeutic response. Dzneladze et al. [146] identified a phosphatase-dependent function for INPP4B in mediating drug response, whereby loss of phosphatase activity ablated the reduced sensitivity to daunorubicin observed with wild-type INPP4B expression. In contrast, Rijal et al. [145] reported that catalytically inactive-INPP4B expression recapitulated the chemoresistant phenotype, suggesting a phosphatase-independent function of INPP4B in cancer. INPP4B expression was not associated with changes in AKT phosphorylation in primary AML samples or in cell lines, further suggesting an AKT-independent function for INPP4B. INPP4B may have more diverse molecular functions beyond its role as a lipid and protein phosphatase, and examination of potential protein–protein interactions may further elucidate its complex and dynamic role in cancer signalling.

INPP4B expression is altered in human cancers and the phosphatase appears to play both oncogenic and tumour suppressor roles depending on whether expression is increased or decreased. These studies suggest a thorough examination of the molecular functions of the inositol polyphosphate phosphatases in different cancers may reveal novel roles beyond their canonical tumour suppressor roles as negative regulators of PI3K/AKT signalling.

## Regulation of phosphoinositide phosphatases by miRNAs

Reduced expression of PI3K pathway enzymes such as PTEN, PIPP and INPP4B is frequently observed in human cancers, which may be due to loss of chromosomal regions, mRNA or protein expression. Small non-coding miRNAs are critical regulators of gene expression including many PI3K pathway members and are frequently dysregulated in human cancers. miRNAs down-regulate mRNA transcripts by binding to the 3′-UTR and promoting degradation of the target mRNA and regulate the majority of the transcriptome. Down-regulation of protein phosphatases PP2A and PHLPP is mediated by miRNAs. *miR222* targets the *PPP2R2A*subunit of PP2A in HCC to disrupt cell motility and *miR-190* inhibits *PHLPP* expression and promotes carcinogenic transformation of bronchial cells suggesting that the AKT pathway is a prominent target of miRNA activity [148,149]. In addition, expression of phosphoinositide phosphatases such as PTEN, PIPP and INPP4B is modulated by miRNAs. In human cancers, *PTEN*is frequently targeted by miRNAs such as *miR21, miR22, miRN214* and *miR221* [150]. *miR21* promotes *PTEN*-transcript degradation and enhances tumour growth, and is frequently up-regulated in colorectal, ovarian and triple negative breast cancers [151–153]. On the other hand, genomic loss of *miR-494* or *miR-599* promotes increased *INPP4B* expression in melanoma cells, and introduction of antisense-miRNA oligonucleotides targeted to *miR494* or *miR599* promotes melanoma cell proliferation through up-regulation of INPP4B [143]. However, miRNA-mediated down-regulation of multiple phosphoinositide phosphatases such as PIPP and PTEN may be characteristic of some cancer cells. Expression of *miR-3127* and *miR-508* is frequently increased in some human cancers suppressing expression of *PIPP* as well as other PI3K/AKT signalling pathway components including *PTEN, INPP4A* and *PHLPP1/2* [154,155]. Exogenous expression of *miR-508* increased and inhibition decreased cell proliferation, colony formation and anchorage-independent cell growth of oesophageal squamous cell carcinoma (ESCC) cells [155]. Similarly, up-regulation of *miR-3127* resulted in increased anchorage-independent cell growth in HCC cells [154]. The ability of single miRNAs to regulate the expression of multiple phosphoinositide phosphatases may have cumulative effects on PI3K/AKT signalling, and given miRNA-targeted therapies are emerging in clinical development it may also provide a more robust treatment strategy with the ability to alter expression of multiple phosphatases simultaneously.

## Conclusions

The inositol polyphosphate phosphatases are dysregulated in many human cancers, and although they were originally predicted to be negative regulators of canonical PI3K/AKT signalling, recent studies suggest they may play more complex roles in tumorigenesis [128, 143, 144, 145, 146]. The spatio-temporal regulation of phosphoinositide pools by the phosphatases creates dynamic signalling gradients that are critical for the maintenance of signalling homoeostasis and when disrupted may lead to cellular transformation. Ablation of these phosphatases can drive oncogenic PI3K signalling through distinct AKT isoform-dominant effectors, leading to diverse phenotypic outcomes. The relative expression levels of AKT1 compared with AKT2 may mediate this distinction, where AKT1 is the primary effector in response to phosphatase ablation except in tissues such as the thyroid where AKT2 is more highly expressed. However, AKT2-addicted phenotypes observed in *PTEN*-depleted cancer cells suggest that alternate regulatory mechanisms may be at play. AKT isoforms have distinct subcellular localizations and thus it is conceivable that compartmentalization of the lipid phosphatases with enriched pools of distinct AKT isoforms may contribute to their downstream function. The activation states of AKT isoforms may also contribute to this divergence, and the activity of the various AKT kinases and phosphatases together with the phosphoinositide phosphatases may affect the activation and function of the AKT isoforms.

Further investigation of phosphatase-independent functions and the cumulative effects of multiple phosphatase dysregulation within the same cancers may also help define the highly dynamic and complex roles these phosphatases play in tumorigenesis and enable determination of whether PI3K and/or AKT inhibitor treatments, or alternative approaches will be effective therapeutic cancer strategies. The PI3Kδ-inhibitor idelalisib is approved for patient use in chronic lymphocytic leukaemia, small lymphocytic lymphoma and follicular lymphoma, and several other PI3K inhibitors are undergoing phase II–III trials [156]. However, clinical trials with AKT inhibitors have shown limited clinical success, and miltefosine is currently the only approved therapy as a topical treatment for cutaneous breast cancer [157]. AKT remains a problematic therapeutic target given the sequence homology among isoforms especially in the kinase domain, but also its structural similarity to other kinase families such as the AGC kinases. Additionally, given the opposing functions of AKT isoforms in some cancers, inhibition of pan-AKT has the potential for undesired physiological consequences on tumour growth and metastasis in cancers such as glioma, breast cancer and vascular tumours. It is therefore possible that AKT isoform-specific inhibitors, perhaps targeted to the more unique regulatory or PH domains, may prove a more viable therapeutic strategy especially in patients with phosphatase dysregulation. Given the distinct functional roles of the inositol polyphosphate phosphatases on AKT isoform signalling and the potential oncogenic effects of phosphatases such as INPP4B, examination of the lipid phosphatase signalling networks dysregulated in specific cancers may identify novel drug targets or therapeutic approaches.

## Abbreviations

AML: acute myeloid leukaemia
DNA-PK: DNA-dependent protein kinase
EEA1: early endosome antigen 1
EGFR: epidermal growth factor receptor
ER: oestrogen receptor
FOXO: forkhead box O
FV-PTC: follicular variant papillary thyroid carcinoma
GPCR: G-protein-coupled receptor
GSK3β: glycogen synthase kinase 3 beta
HCC: hepatocellular carcinoma
HDAC2: histone deacetylase 2
IGF-1: insulin-like growth factor 1
ILK: integrin-linked kinase
INPP4B: inositol polyphosphate 4-phosphatase type II
INPP5J: inositol polyphosphate 5-phosphatase J
Ins(3,4)*P*_2_: inositol-3,4-bisphosphate
LOH: loss of heterozygosity
MMTV: mouse mammary tumour virus
mTORC: mammalian target of rapamycin complex
NFAT: nuclear factor of activated T cells
PDFGB: platelet-derived growth factor subunit B
PDK1: phosphoinositide-dependent kinase 1
PH: pleckstrin homology
PHLPP: PH domain and leucine-rich repeat protein phosphatase
PI3K: phosphoinositide 3-kinase
PIPP: proline-rich inositol polyphosphate 5-phosphatase
PP2A: protein phosphatase 2
PRAS40: proline-rich AKT substrate of 40 kDa
PtdIns3*P*: phosphatidylinositol 3-phosphate
PtdIns(3,4)*P*_2_: phosphatidylinositol 3,4-bisphosphate
PtdIns(3,4,5)*P*_3_: phosphatidylinositol 3,4,5-trisphosphate
PtdIns(4,5)*P*_2_: phosphatidylinositol 4,5-bisphosphate
PTEN: phosphatase and tensin homologue
PyMT: polyoma virus middle T antigen
RCAS-TVA: replication-competent avian sarcoma-leukosis virus long terminal repeat with splice acceptor tumour virus A
RTK: receptor tyrosine kinase
SGK3: serum and glucocorticoid-regulated kinase
SKICH: SKIP carboxyl homology
